# Supplementary material for: Mental health symptoms as preclinical indicators of dementia: a Whitehall II cohort study
Source: medRxiv. 2026 Jan 8:2026.01.08.26343594. Preprint. [Version 1] doi: 10.64898/2026.01.08.26343594 (PMC12803369; doi:10.64898/2026.01.08.26343594)
Supplement: Supplement 1 [file media-1.pdf]

SUPPLEMENT

Supplementary methods

Description of inverse probability weighting procedure

We firstly used logistic regression to estimate the probability of being in the sample (i.e. of having completed the CIS-R in 2012-13), using the following demographic, lifestyle, and health-related variables: age, ethnicity, sex, marital status, education, fruit and vegetable intake, physical activity, diabetes, hypertension, multimorbidity, depression, other mental health disorder, and death during follow-up. There were a few participants missing data on the above variables, so we did a single imputation to impute the missing values. We assessed various interaction terms between demographic variables and all other variables by stepwise selection (alpha <0.05) to create a model that best predicted being in the sample. The inverse of the estimated probabilities was used as weights in the adjusted Cox regression model.

Table 1. Definition of clinical conditions ascertained from linked records

**Condition**	**Hospital Episode Statistics data ICD-10 (-9) codes [exclusions]**	**Whitehall II Records (results from previous phases)**	**Mental Health Services Dataset**	**Mortality Records**	**Cancer Registry**
Cancer	CXX [C44]				Same ICD codes
Chronic kidney disease	N18 (585)				
Chronic obstructive pulmonary disease	J41, J42, J43, J44 (491, 492, 496)				
Coronary heart disease	I20, I21, I22, I23, I24, I25 (410, 411, 412, 413, 414)	12 lead resting electrocardiogram recording		Same ICD codes	
Dementia	F00-03, F05.1, G30-31		Same ICD codes	Same ICD codes	
Diabetes	E11 (250)	Report by doctor, use of diabetic drugs,<br>fasting glucose ≥7mmol/L, longstanding illness questionnaire			
Heart failure	I50 (428)				
Hypertension	I10, I11, I12, I13, I15 (401, 402, 403, 404, 405)	Blood pressure measures, use of antihypertensive drugs			
Liver disease	K7X (571)				
Parkinson's	G20 (332)			Same ICD codes	
Rheumatoid arthritis	M05, M06				
Stroke	I60, I61, I63, I64 (430, 431, 434, 436)	MONICA Ausburg stroke questionnaire [48] (Phases 1-9)		Same ICD codes	
Osteoarthritis	M15, M16, M17, M18, M19 (715)				

ICD = International Classification of Diseases

Figure 1. Study population flow chart

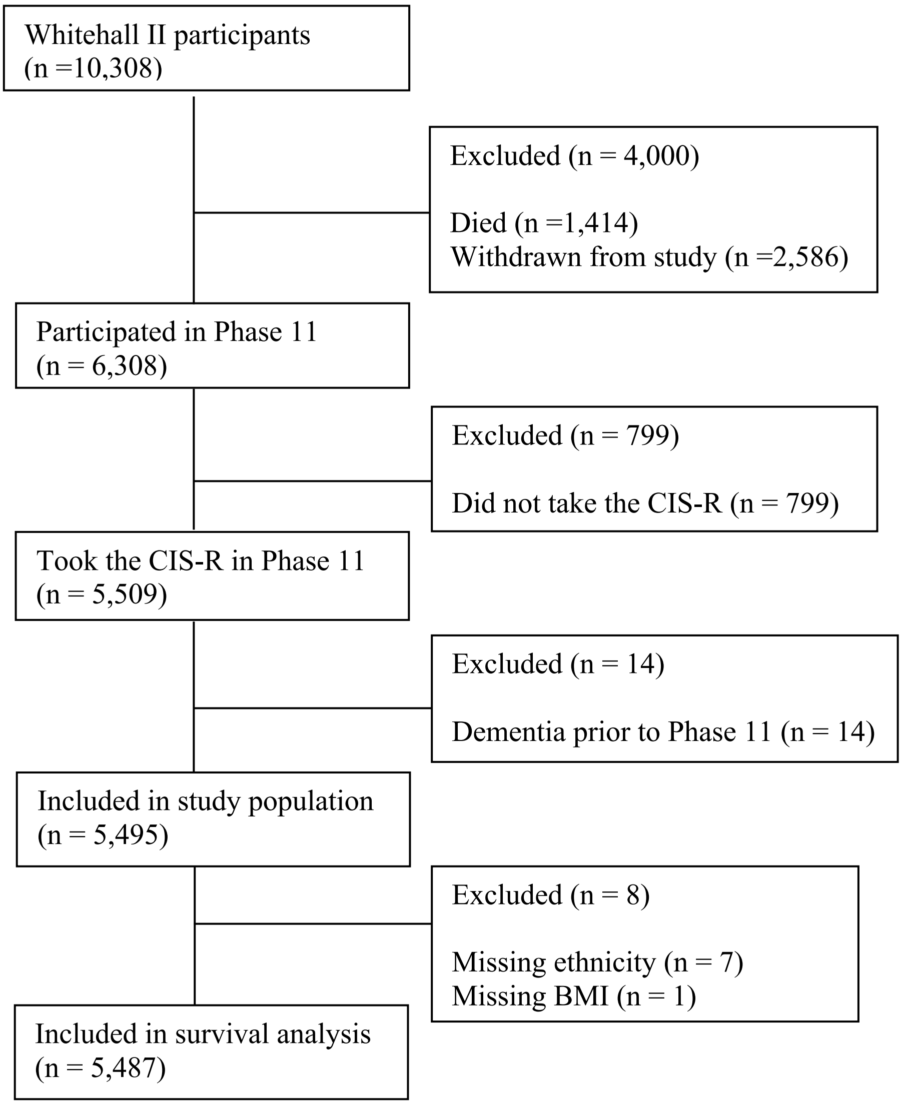

BMI= Body mass index; CIS-R=Clinical Interview Schedule – Revised

Table 2. Prevalence of mental health symptoms (overall and each subscore) at 2015-16, in the full study population and by dementia status at the end of follow-up

Characteristics	Overall N = 4,609	No dementia N=4,395	Dementia N = 314
Follow-up time (in years), median (IQR)	8.03 (7.52, 8.54)	8.09 (7.59, 8.57)	4.87 (2.27, 6.36)
Median total CIS-R score (IQR)	2 (0, 5)	2 (0, 4)	3 (1, 6)
Any mental health condition (total CIS-R score ≥12)	372 (8,1%)	334 (7.8%)	38 (12.1%)
Severe mental health condition (total CIS-R score ≥18)	169 (3.7%)	149 (3.5%)	20 (6.4%)
Symptoms (CIS-R subscore ≥2)			
Sleep problem symptoms	1,015 (22.0%)	960 (21.8%)	55 (17.5%)
Fatigue symptoms	866 (18.8%)	785 (18.3%)	81 (25.8%)
Irritability symptoms	335 (7.3%)	301 (7.0%)	34 (10.8%)
Worry symptoms	389 (8.4%)	358 (8.3%)	31 (9.9%)
Concentration symptoms	366 (7.9%)	298 (6.9%)	68 (21.7%)
Obsessions symptoms	265 (5.7%)	245 (5.7%)	20 (6.4%)
Depression symptom	265 (5.7%)	230 (5.4%)	35 (11.1%)
Somatic symptoms	277 (6.0%)	250 (5.8%)	27 (8.6%)
Depressive ideas symptoms	208 (4.5%)	183 (4.3%)	25 (8.0%)
Anxiety symptoms	174 (3.8%)	157 (3.7%)	17 (5.4%)
Compulsive behaviour symptoms	160 (3.5%)	138 (3.2%)	22 (7.0%)
Worry over physical health symptoms	180 (3.9%)	161 (3.7%)	19 (6.1%)
Phobia symptoms	60 (1.3%)	53 (1.2%)	7 (2.2%)
Panic symptoms	36 (0.8%)	34 (0.8%)	2 (0.6%)

CIS-R = Clinical Interview Schedule – Revised; IQR = Interquartile Range

Table 3a. Association between any mental health condition (total CIS-R score ≥ 12) and severe mental health condition (total CIS-R score ≥18) and dementia risk at each year of follow-up

Year of follow-up	Risk of dementia[a]	Any mental health condition Adjusted HR (95% CI)	Severe mental health condition Adjusted HR (95% CI)
1	13/5,495=0.24%	1.91 (0.65, 5.96)	2.62 (0.63, 11.0)
2	23/5,458=0.42%	4.03 (2.38, 6.82)	4.73 (2.39, 9.36)
3	21/5,393=0.39%	4.04 (2.52, 6.50)	4.73 (2.54, 8.81)
4	37/5,316=0.70%	3.11 (2.02, 4.79)	3.79 (2.14, 6.72)
5	42/5,212=0.81%	1.04 (1.30, 3.22)	2.69 (1.49, 4.85)
6	47/5,104=0.92%	1.26 (0.63, 2.49)	1.78 (0.76, 4.16)
7	37/4,991=0.74%	0.97 (0.48, 1.93)	1.29 (0.52, 3.24)
8	44/4,897=0.90%	0.93 (0.57, 1.53)	1.07 (0.50, 2.25)
9	49/4,778=1.03%	1.00 (0.63, 1.57)	0.97 (0.47, 2.02)
10	58/4,642=1.25%	1.04 (0.59, 1.83)	0.90 (0.35, 2.33)
11	62/4,481=1.38%	1.02 (0.52, 2.00)	0.82 (0.26, 2.56)
12.1	22/3,703=0.59%	0.97 (0.46, 2.05)	0.75 (0.22, 2.59)

CI = confidence interval; HR = hazard ratio

[a]Numerator: Number of participants with dementia between Y-1 and Y; Denominator: Number of participants at risk for dementia at Y-1

Hazard ratios and their confidence intervals were estimated using flexible parametric models adjusted for age, sex, ethnicity, marital status, education, fruit and vegetable intake, physical activity, alcohol consumption, smoking, BMI, diabetes, hypertension, and multimorbidity.

Table 3b. Association between mental health symptoms (CIS-R subscore ≥ 2) and dementia risk at each year of follow-up

Year of follow-up	Risk of dementia[a]	Somatic Adjusted HR (95% CI)	Fatigue Adjusted HR (95% CI)	Concentration problems Adjusted HR (95% CI)	Sleep problems Adjusted HR (95% CI)
1	13/5,495=0.24%	1.28 (0.35, 4.67)	2.43 (0.64, 3.19)	3.49 (1.54, 7.90)	0.79 (0.30, 2.09)
2	23/5,458=0.42%	1.91 (0.95, 3.85)	2.07 (1.28, 3.35)	5.31 (3.31, 8.53)	1.43 (0.87, 2.36)
3	21/5,393=0.39%	1.90 (1.01, 3.55)	2.09 (1.37, 3.18)	5.06 (3.31, 7.73)	1.57 (1.02, 2.42)
4	37/5,316=0.70%	1.63 (0.95, 2.81)	1.85 (1.30, 2.65)	4.10 (2.84, 5.91)	1.43 (0.99, 2.08)
5	42/5,212=0.81%	1.31 (0.76, 2.24)	1.55 (1.10, 2.18)	3.05 (2.12, 4.41)	1.20 (0.85, 1.69)
6	47/5,104=0.92%	1.04 (0.52, 2.09)	1.28 (0.85, 1.94)	2.23 (1.37, 3.61)	0.98 (0.64, 1.49)
7	37/4,991=0.74%	0.96 (0.50, 1.84)	1.14 (0.76, 1.71)	1.81 (1.11, 2.93)	0.85 (0.56, 1.29)
8	44/4,897=0.90%	0.98 (0.60, 1.60)	1.08 (0.77, 1.50)	1.62 (1.10, 2.40)	0.79 (0.56, 1.11)
9	49/4,778=1.03%	1.06 (0.67, 1.66)	1.07 (0.78, 1.46)	1.56 (1.06, 2.28)	0.76 (0.56, 1.04)
10	58/4,642=1.25%	1.21 (0.63, 1.99)	1.05 (0.70, 1.57)	1.50 (0.92, 2.45)	0.74 (0.50, 1.11)
11	62/4,481=1.38%	1.15 (0.57, 2.32)	1.03 (0.63, 1.68)	1.42 (0.78, 2.57)	0.72 (0.44, 1.18)
12.1	22/3,703=0.59%	1.15 (0.51, 2.57)	0.99 (0.57, 1.73)	1.33 (0.68, 2.58)	0.69 (0.40, 1.21)

CI = confidence interval; HR = hazard ratio

[a]Numerator: Number of participants with dementia between Y-1 and Y; Denominator: Number of participants at risk for dementia at Y-1

Hazard ratios and their confidence intervals were estimated using flexible parametric models adjusted for age, sex, ethnicity, marital status, education, fruit and vegetable intake, physical activity, alcohol consumption, smoking, BMI, diabetes, hypertension, and multimorbidity.

Table 3b (continued). Association between mental health symptoms (CIS-R subscore ≥ 2) and dementia risk at each year of follow-up

Year of follow-up	Risk of dementia[a]	Irritability Adjusted HR (95% CI)	Worry over physical health Adjusted HR (95% CI)	Depressive ideas Adjusted HR (95% CI)	Worry Adjusted HR (95% CI)
1	13/5,495=0.24%	1.16 (0.27, 5.07)	1.07 (0.16, 7.32)	2.33 (0.66, 8.20)	1.31 (0.37, 4.71)
2	23/5,458=0.42%	2.87 (1.54, 5.35)	2.70 (1.25, 5.84)	4.32 (2.37, 7.88)	2.27 (1.20, 4.29)
3	21/5,393=0.39%	3.29 (1.92, 5.65)	2.94 (1.48, 5.86)	4.13 (2.36, 7.23)	2.37 (1.34, 4.20)
4	37/5,316=0.70%	2.85 (1.75, 4.63)	2.32 (1.19, 4.53)	3.08 (1.82, 5.19)	2.07 (1.26, 3.39)
5	42/5,212=0.81%	2.11 (1.32, 3.37)	1.50 (0.75, 2.98)	1.95 (1.12, 3.42)	1.64 (1.02, 2.64)
6	47/5,104=0.92%	1.44 (0.74, 2.81)	0.86 (0.30, 2.51)	1.17 (0.50, 2.75)	1.27 (0.68, 2.38)
7	37/4,991=0.74%	1.09 (0.53, 2.25)	0.65 (0.21, 2.00)	0.97 (0.43, 2.18)	1.10 (0.59, 2.05)
8	44/4,897=0.90%	0.95 (0.54, 1.66)	0.64 (0.28, 1.47)	1.04 (0.59, 1.84)	1.05 (0.65, 1.71)
9	49/4,778=1.03%	0.91 (0.54, 1.51)	0.72 (0.34, 1.52)	1.22 (0.73, 2.05)	1.07 (0.69, 1.67)
10	58/4,642=1.25%	0.87 (0.45, 1.70)	0.77 (0.31, 1.87)	1.34 (0.72, 2.51)	1.09 (0.62, 1.90)
11	62/4,481=1.38%	0.82 (0.37, 1.84)	0.76 (0.27, 2.14)	1.37 (0.66, 2.86)	1.07 (0.54, 2.11)
12.1	22/3,703=0.59%	0.76 (0.31, 1.88)	0.73 (0.23, 2.29)	1.34 (0.59, 3.06)	1.04 (0.48, 2.25)

CI = confidence interval; HR = hazard ratio

[a]Numerator: Number of participants with dementia between Y-1 and Y; Denominator: Number of participants at risk for dementia at Y-1

Hazard ratios and their confidence intervals were estimated using flexible parametric models adjusted for age, sex, ethnicity, marital status, education, fruit and vegetable intake, physical activity, alcohol consumption, smoking, BMI, diabetes, hypertension, and multimorbidity.

Table 3b (continued). Association between mental health symptoms (CIS-R subscore ≥ 2) and dementia risk at each year of follow-up

Year of follow-up	Risk of dementia[a]	Anxiety Adjusted HR (95% CI)	Compulsions Adjusted HR (95% CI)	Obsessions Adjusted HR (95% CI)	Depression Adjusted HR (95% CI)
1	13/5,495=0.24%	0.79 (0.10, 6.25)	2.09 (0.74, 5.88)	0.85 (0.24, 2.96)	1.39 (0.35, 5.61)
2	23/5,458=0.42%	2.95 (1.36, 6.39)	1.93 (0.84, 4.40)	0.82 (0.31, 2.15)	3.53 (1.97, 6.35)
3	21/5,393=0.39%	3.79 (2.08, 6.90)	1.84 (0.94, 3.62)	0.85 (0.40, 1.80)	3.76 (2.23, 6.33)
4	37/5,316=0.70%	3.26 (1.84, 5.78)	1.79 (1.04, 3.08)	0.90 (0.50, 1.63)	2.87 (1.75, 4.71)
5	42/5,212=0.81%	2.20 (1.22, 3.99)	1.75 (1.01, 3.03)	0.96 (0.54, 1.71)	1.77 (1.06, 2.97)
6	47/5,104=0.92%	1.30 (0.51, 3.28)	1.73 (0.90, 3.34)	1.02 (0.52, 2.01)	0.98 (0.43, 2.24)
7	37/4,991=0.74%	0.99 (0.38, 2.57)	1.76 (0.95, 3.25)	1.03 (0.54, 1.99)	0.77 (0.34, 1.75)
8	44/4,897=0.90%	1.01 (0.53, 1.93)	1.82 (1.12, 2.95)	1.01 (0.59, 1.71)	0.82 (0.47, 1.44)
9	49/4,778=1.03%	1.17 (0.67, 2.03)	1.89 (1.22, 2.94)	0.96 (0.61, 1.53)	0.98 (0.60, 1.60)
10	58/4,642=1.25%	1.28 (0.64, 2.53)	1.96 (1.12, 3.44)	0.92 (0.50, 1.71)	1.09 (0.61, 1.95)
11	62/4,481=1.38%	1.30 (0.57, 2.96)	2.01 (0.99, 4.07)	0.90 (0.41, 1.98)	1.12 (0.56, 2.21)
12.1	22/3,703=0.59%	1.28 (0.51, 3.22)	2.04 (0.90, 4.62)	0.89 (0.35, 2.22)	1.09 (0.51, 2.35)

CI = confidence interval; HR = hazard ratio

[a]Numerator: Number of participants with dementia between Y-1 and Y; Denominator: Number of participants at risk for dementia at Y-1

Hazard ratios and their confidence intervals were estimated using flexible parametric models adjusted for age, sex, ethnicity, marital status, education, fruit and vegetable intake, physical activity, alcohol consumption, smoking, BMI, diabetes, hypertension, and multimorbidity.

Table 4. Association between the CIS-R overall mental health condition score and symptom subscores and risk of dementia in original study population and using inverse probability weighted study population

Condition	Adjusted HR Unweighted (95% CI)	p-value	Adjusted HR Weighted (95% CI)	p-value
Any mental health condition (CIS-R subscore ≥12)	1.63 (1.22, 2.17)	<0.001	1.70 (1.24, 2.33)	<0.001
Severe mental health condition (CIS-R subscore ≥18)	1.91 (1.28, 2.86)	0.002	2.03 (1.32, 3.14)	0.001
Symptom (CIS-R subscore ≥2)				
Somatic	0.84 (0.56, 1.23)	0.40	1.25 (0.85, 1.83)	0.256
Fatigue	1.31 (1.04, 1.66)	0.021	1.28 (1.00, 1.65)	0.051
Concentration	2.46 (1.92, 3.14)	<0.001	2.46 (1.89, 3.20)	<0.001
Sleep	0.95 (0.75, 1.19)	0.60	1.02 (0.80, 1.31)	0.869
Irritability	1.47 (1.07, 2.03)	0.017	1.53 (1.09, 2.15)	0.014
Worry over physical health	1.20 (0.77, 1.87)	0.40	1.27 (0.79, 2.05)	0.326
Depressive ideas	1.82 (1.30, 2.57)	<0.001	2.02 (1.38, 2.96)	<0.001
Worry	1.38 (1.01, 1.88)	0.044	1.32 (0.96, 1.83)	0.090
Anxiety	2.49 (1.27, 4.87)	0.008	1.77 (1.18, 2.65)	0.005
Compulsions	1.90 (1.34, 2.70)	<0.001	1.75 (1.19, 2.59)	0.005
Obsessions	0.94 (0.65, 1.38)	0.80	0.80 (0.53, 1.22)	0.305
Depression	1.51 (1.10, 2.07)	0.012	1.71 (1.21, 2.42)	0.002

HR = Hazard ratio; CI = Confidence interval

Cox regression models adjusted for age, sex, ethnicity, marital status, education, fruit and vegetable intake, physical activity, alcohol consumption, smoking, BMI, diabetes, hypertension, and multimorbidity
